# Imaging findings and clinical outcomes in neonates with isolated subependymal echogenicity on cranial ultrasound: a retrospective cohort study

**DOI:** 10.3389/fnhum.2026.1945002

**Published:** 2026-10-28

**Authors:** Yonghua Xiang, Jingyu Chen, Saifeng Huang, Huiqing Shi, Xiaodong Li, Jian Jiang, Xuehua Zhang

**Affiliations:** 1 The Affiliated Children’s Hospital of Xiangya School of Medicine, Central South University (Hunan Children’s Hospital), Changsha, China; 2 National Clinical Research Center for Children and Adolescents, Health and Diseases, Ministry of Education Key Laboratory of Child Development and Disorders, Chongqing Municipal Health Commission Key Laboratory of Children’s Vital Organ Development and Diseases, Department of Ultrasound, Children’s Hospital of Chongqing Medical University, Chongqing, China; 3 Fujian Maternity and Child Health Hospital, College of Clinical Medicine for Obstetrics & Gynecology and Pediatrics, Fujian Medical University, Fuzhou, China; 4 Department of Neurology, Lingling Branch of Yongzhou Central Hospital, Yongzhou, China

**Keywords:** cerebral palsy, clinical outcome, cranial ultrasonography, magnetic resonance imaging, neonate, subependymal echogenicity, subependymal hemorrhage

## Abstract

**Objective:**

To describe clinical and imaging findings in neonates with isolated subependymal echogenicity on cranial ultrasound and examine associations with recorded mortality and cerebral palsy.

**Methods:**

This retrospective single-center cohort included 317 neonates admitted between January 2015 and December 2018 with an initial ultrasound finding suspicious for isolated subependymal hemorrhage. Clinical records, available MRI findings, and follow-up information were reviewed. Cerebral palsy was ascertained from rehabilitation or neurology diagnostic records, supplemented by telephone follow-up in January 2026, independently of developmental test scores. The primary adjusted analysis compared death with all survivors using an exploratory six-item comorbidity count and clinical covariates; hospitalization duration was excluded, and laterality was coded as unilateral or bilateral.

**Results:**

There were 52 deaths (16.4%), 9 survivors with recorded cerebral palsy (2.8%), and 256 survivors without recorded cerebral palsy (80.8%). MRI findings were compatible with hemorrhage in 61 of 90 examined infants (67.8%). In the complete-case mortality model (*n* = 316), each additional comorbidity was associated with greater odds of death (adjusted OR, 2.462; 95% CI, 1.787–3.391; *p* < 0.001). The other included covariates were not statistically significant. White matter abnormalities were recorded in nine infants, including eight of the nine infants with cerebral palsy and one without recorded cerebral palsy.

**Conclusion:**

Systemic comorbidity burden was associated with mortality within this ultrasound-defined cohort. White matter abnormalities accompanied most recorded cerebral palsy cases, but this observation remains descriptive. Specialist diagnostic records and later telephone follow-up characterize persistent clinical diagnoses; they do not establish the independent effect of subependymal echogenicity or normal development across all domains in survivors without cerebral palsy.

## Introduction

Subependymal echogenicity on cranial ultrasound has long been described in neonates, particularly those born preterm (Moradi et al., 2024; Dolfin et al., 1983; Ahmann et al., 1980; Dolfin et al., 1984). Hemorrhage confined to the germinal matrix without intraventricular extension is generally termed grade I germinal matrix hemorrhage (Atienza-Navarro et al., 2020; Tuburan, 2021; Mohammad et al., 2021). This anatomical diagnosis should be distinguished from the ultrasound appearance of subependymal echogenicity, which may also reflect residual germinal matrix tissue or other nonhemorrhagic changes (Lekic et al., 2015; Ní Leidhin et al., 2025; Indrakanti et al., 2022). Thus, an echogenic lesion is not, by itself, proof of hemorrhage.

Recent neonatal imaging literature emphasizes the differential diagnosis of caudothalamic groove echogenicity and the complementary role of MRI, including susceptibility-sensitive sequences (Ní Leidhin et al., 2025; Yazol et al., 2026). Small lesions can evolve between ultrasound and MRI, and the modalities may depict different stages of the process (Fichera et al., 2024). Accordingly, MRI findings obtained later cannot be used uncritically to label an initial ultrasound examination as true or false positive.

Clinical outcomes after preterm birth are influenced by cerebral immaturity, white matter vulnerability, vascular regulation, and systemic disease (Song, 2023; Brew et al., 2014; Vesoulis and Mathur, 2017; Ostrem and Gano, 2025; McQuillen et al., 2010; Ng et al., 1998; Serdar et al., 2025; Fanconi and Duc, 1987). These conditions may accompany an echogenic subependymal lesion and complicate attribution of adverse outcomes to that lesion. An observational cohort can describe such associations but cannot establish the independent effect of the imaging finding without an appropriate comparison group.

We examined clinical characteristics across gestational age groups, described available MRI findings after an initial ultrasound finding of isolated subependymal echogenicity, and evaluated associations with recorded death. Cerebral palsy and recorded white matter abnormalities were analyzed descriptively because of the small number of events.

## Materials and methods

### Study design and patient selection

This single-center retrospective cohort comprised neonates admitted to Hunan Children’s Hospital between January 2015 and December 2018 with isolated subependymal echogenicity on their initial cranial ultrasound examination. The entry finding was a focal or patchy subependymal echogenic lesion interpreted as suspicious for hemorrhage, without concurrent intraventricular or intraparenchymal hemorrhage, hydrocephalus, major congenital brain malformation, or another structural intracranial abnormality. Isolated refers to the initial examination and does not exclude abnormalities detected subsequently. This ultrasound-defined phenotype is not equivalent to universally confirmed hemorrhage.

Eligibility required admission within 28 days of birth, the specified initial ultrasound finding, and an available recorded outcome. Exclusion criteria included congenital brain malformations, confirmed genetic or metabolic disorders, neurological injury attributable to congenital infection, and other intracranial lesions on initial imaging.

The Ethics Committee of Hunan Children’s Hospital approved the study (Approval No. HCHLL-2026-258). The requirement for informed consent was waived because of the retrospective design and use of de-identified clinical data.

### Gestational age classification

Descriptive groups were derived from gestational age at birth: <30 weeks, 30–36 weeks, and ≥37 weeks. These study-specific strata comprised 58, 174, and 85 infants, respectively. Two inconsistent pre-existing group codes were replaced by groups derived from the continuous gestational age values.

### Cranial ultrasonography

Bedside cranial ultrasonography was performed within 72 h of admission using a Mindray M9 system and a 5–12 MHz transducer. Coronal and sagittal views were acquired through the anterior fontanelle, with additional mastoid or posterior fontanelle views when indicated. Follow-up scans were obtained at intervals of 5–7 days according to clinical stability. The initial examination provided baseline lesion characteristics; follow-up examinations were reviewed for evolution. Two pediatric sonographers independently reviewed the images while blinded to outcomes, with disagreements resolved with a senior pediatric radiologist. Reader-level ratings were unavailable for calculating interobserver agreement.

### Brain MRI

MRI was obtained when clinically indicated in infants stable enough to undergo examination and with parental consent. Examinations used a 3.0-T scanner with T1-weighted, T2-weighted, fluid-attenuated inversion recovery, and diffusion-weighted sequences. Susceptibility-weighted or T2*-weighted gradient-echo images were reviewed when available. Two pediatric neuroradiologists interpreted the examinations and resolved discrepancies by consensus. MRI findings were described within the selected subgroup.

White matter abnormalities were summarized as a broad imaging category. Cystic periventricular leukomalacia (PVL), punctate white matter lesions, and persistent periventricular echogenicity have distinct imaging characteristics and should not be treated as interchangeable diagnoses (Mohammad et al., 2021; Ní Leidhin et al., 2025; Dudink et al., 2020). Although the original database field was labeled PVL, it was interpreted here as a record of white matter abnormalities. Lesion-specific criteria and timing were not consistently available for subtype classification; the pooled category therefore does not imply that all positive records represented cystic PVL.

### Clinical data collection

Variables included gestational age, birth weight, sex, delivery mode, singleton or multiple birth, lesion laterality, hospitalization duration, and neonatal complications. Left- and right-sided lesions were combined as unilateral; bilateral lesions formed the comparison category. Birth weight was recorded in grams and scaled per 100 g in regression models. One value of 0 g was treated as invalid and set to missing in the analysis copy, without imputing a replacement value or changing the source file.

The exploratory comorbidity count assigned one point each for respiratory distress syndrome (RDS), sepsis, disseminated intravascular coagulation (DIC), congenital heart disease (CHD), necrotizing enterocolitis (NEC), and multiple organ failure (MOF), for a possible total of 0–6. The stored total matched the sum of the six indicators for all 317 infants. Equal weighting does not account for severity, timing, duration, or overlap between conditions, and the score is not externally validated.

### Follow-up and outcome assessment

Clinical outcomes were classified as death, survival with recorded cerebral palsy, or survival without recorded cerebral palsy. Deaths were retained in the mortality analysis and excluded from the description of cerebral palsy among survivors. Cerebral palsy was ascertained from diagnoses documented in rehabilitation or neurology records, supplemented by telephone verification of an established clinical diagnosis. The clinical definition of cerebral palsy encompasses lasting impairment of movement and posture that restricts everyday activities and results from a non-progressive disturbance of the developing fetal or infant brain (Rosenbaum et al., 2007). Its recognition depends on clinical neurological assessment of the motor disorder and its developmental course. Developmental test scores alone do not establish this diagnosis. The Gross Motor Function Classification System (GMFCS) describes gross motor function after diagnosis and was not used as a diagnostic criterion (Richards and Malouin, 2013). Individual ages at diagnosis and the identities of the diagnosing clinicians were not available for retrospective abstraction.

Bayley Scales of Infant and Toddler Development, Third Edition (Bayley-III), assessments had been performed during follow-up and provided supplementary information on early development (Balasundaram and Avulakunta, 2022; Robertson, 2010). Cognitive Composite <85 or Motor Composite <85 are score-based criteria for developmental abnormality, which must be distinguished from a clinical cerebral palsy diagnosis. Bayley-III scores were not used to adjudicate cerebral palsy or to define the outcome groups in the present analysis. MDI/PDI and Neonatal Behavioral Neurological Assessment (NBNA) entries represented separately collected early assessments and were likewise not used for outcome classification. Survival without recorded cerebral palsy was therefore not equated with normal development across all assessed or unassessed domains.

Supplementary telephone follow-up was conducted in January 2026 to verify persistent clinically diagnosed conditions, including cerebral palsy, and to update clinical status in relation to previous records. Telephone contact was not used to make a new neurological diagnosis or to replace an in-person developmental assessment. The date of telephone contact was distinguished from the date and age of formal developmental testing.

### Statistical analysis

The revised analyses used Python 3.12 with SciPy and statsmodels. Continuous and ordinal variables were summarized as median (interquartile range) and compared using tie-corrected Kruskal–Wallis tests. Categorical variables were summarized as *n* (%) using column denominators. Pearson *χ*^2^ tests were used when all expected cell counts were at least 5; otherwise, Fisher–Freeman–Halton tests were used. Exact probabilities were enumerated for 2 × 3 tables. The 3 × 3 gestational-age-by-outcome table used 200,000 fixed-margin Monte Carlo samples with seed 20,260,927. *p* values were two-sided, with 0.05 as the nominal threshold. Univariate comparisons were exploratory and were not used to screen covariates for the adjusted model.

The primary adjusted analysis used binary logistic regression for death versus all survivors, including those with cerebral palsy. The seven covariates were delivery mode, birth weight per 100 g, gestational age per week, sex, multiple birth, comorbidity count, and bilateral versus unilateral echogenicity. This set was specified for the revision to retain the clinical domains in the updated analysis, without claiming prospective prespecification. Hospitalization duration was excluded because it can be shortened by death. The complete-case model included 316 infants (52 deaths and 264 survivors); the one infant with invalid birth weight was excluded only from analyses requiring this variable. Adjusted ORs and Wald 95% CIs were reported.

A smaller sensitivity model included gestational age, comorbidity count, and laterality (*n* = 317), reducing parameter burden and avoiding simultaneous adjustment for gestational age and birth weight. The full model converged, and the largest variance inflation factor was 3.54; these checks do not establish predictive validity or eliminate overfitting. The updated multinomial model was also recomputed for comparison (Supplementary Table S1), but its cerebral palsy estimates were not used for confirmatory inference because only nine events were available. Neither logistic formulation models event times or resolves the fact that death prevents later cerebral palsy ascertainment; no competing-risk cumulative incidence was estimated.

## Results

### Baseline clinical characteristics of the study cohort

The cohort included 317 infants, comprising 201 males (63.4%) and 116 females (36.6%); 52 infants (16.4%) were from multiple births. Gestational age ranged from 25 to 41 weeks, with a median of 33 (IQR, 30–37). After excluding one invalid 0-g entry, birth weight ranged from 660 to 5,210 g (*n* = 316), with a median of 1,850 g (IQR, 1,300–2,500). Median hospitalization duration was 30 days (IQR, 17–53). RDS occurred in 165 infants (52.1%), sepsis in 110 (34.7%), DIC in 99 (31.2%), CHD in 100 (31.5%), NEC in 68 (21.5%), and MOF in 5 (1.6%). The median comorbidity count was 2 (IQR, 1–3).

### Clinical characteristics according to gestational age

After consistent derivation from continuous gestational age, the <30-week, 30–36-week, and ≥37-week groups included 58, 174, and 85 infants. Birth weight, hospitalization duration, and comorbidity count differed across groups (all *p* < 0.001). Multiple birth, delivery mode, RDS, sepsis, DIC, CHD, and laterality also differed across groups, while sex, NEC, and MOF did not reach the nominal significance threshold (Table 1). These are overall comparisons and do not establish every pairwise difference.

**Table 1:** Clinical characteristics by derived gestational age group.

Characteristic	<30 weeks (*n* = 58)	30–36 weeks (*n* = 174)	≥37 weeks (*n* = 85)	*p*
Birth weight, *g*	1,010 (905–1,200)	1,820 (1,450–2,150)	2,900 (2,390–3,240)	<0.001ᵃ
Hospitalization, days	72 (53–84)	35.5 (24.25–50)	14 (9–23)	<0.001ᵃ
Comorbidity count	3 (2–3.75)	2 (1–2)	1 (0–1)	<0.001ᵃ
Male	38 (65.5)	102 (58.6)	61 (71.8)	0.111ᵇ
Vaginal delivery	48 (82.8)	81 (46.6)	37 (43.5)	<0.001ᵇ
Multiple birth	16 (27.6)	32 (18.4)	4 (4.7)	<0.001ᵇ
Bilateral echogenicity	25 (43.1)	119 (68.4)	46 (54.1)	0.001ᵇ
Respiratory distress syndrome	47 (81.0)	101 (58.0)	17 (20.0)	<0.001ᵇ
Sepsis	35 (60.3)	57 (32.8)	18 (21.2)	<0.001ᵇ
Disseminated intravascular coagulation	31 (53.4)	59 (33.9)	9 (10.6)	<0.001ᵇ
Congenital heart disease	29 (50.0)	47 (27.0)	24 (28.2)	0.004ᵇ
Necrotizing enterocolitis	12 (20.7)	40 (23.0)	16 (18.8)	0.736ᵇ
Multiple organ failure	2 (3.4)	1 (0.6)	2 (2.4)	0.148ᶜ

Values are median (IQR) or *n* (%). Groups were derived from continuous gestational age; two stored group codes were inconsistent. Birth weight *n* = 58, 173, and 85 because one 0-g value was excluded. Other displayed variables have complete data. Hospitalization is descriptive only and is excluded from regression. ᵃKruskal–Wallis; ᵇPearson *χ*^2^; ᶜFisher–Freeman–Halton exact. All categorical rows show the indicated category versus its complement. Overall tests do not imply pairwise differences.

### MRI findings after ultrasound diagnosis

The initial subependymal echogenic lesions were unilateral in 127 infants (40.1%; left, 72; right, 55) and bilateral in 190 (59.9%). MRI was obtained in 90 infants (28.4% of the cohort), of whom 61 (67.8%) had findings compatible with hemorrhage and 29 (32.2%) did not. The interquartile range of the interval between the initial ultrasound examination and MRI was 1–30 days. These findings describe a selected subgroup and do not estimate ultrasound sensitivity, specificity, or overall diagnostic agreement.

### Recorded clinical outcomes

There were 52 recorded deaths (16.4%), 9 survivors with cerebral palsy (2.8%), and 256 survivors without recorded cerebral palsy (80.8%). The recorded cerebral palsy proportion among survivors was 9/265 (3.4%); this is not a competing-risk cumulative incidence. One cerebral palsy case occurred in the <30-week group and eight in the 30–36-week group; none occurred in the ≥37-week group. The three-category outcome distribution differed across the derived gestational age groups (Monte Carlo exact *p* = 0.002; Table 2).

**Table 2:** Unadjusted comparisons across recorded clinical outcome groups.

Characteristic	Death (*n* = 52)	Cerebral palsy (*n* = 9)	Survival without recorded CP (*n* = 256)	*p*
Birth weight, *g*	1,695 (1,022.5–2,325)	1,450 (1,290–1,450)	1,900 (1,355–2,542.5)	0.025ᵃ
Gestational age, weeks	33 (28–37)	31 (31–32)	34 (31–37)	0.189ᵃ
Comorbidity count	2.5 (2–4)	2 (1–2)	1 (1–2)	<0.001ᵃ
Gestational age <30 weeks	17 (32.7)	1 (11.1)	40 (15.6)	0.002ᵈ
Gestational age 30–36 weeks	18 (34.6)	8 (88.9)	148 (57.8)	–
Gestational age ≥37 weeks	17 (32.7)	0 (0.0)	68 (26.6)	–
Male	38 (73.1)	8 (88.9)	155 (60.5)	0.070ᶜ
Vaginal delivery	28 (53.8)	3 (33.3)	135 (52.7)	0.544ᶜ
Multiple birth	6 (11.5)	4 (44.4)	42 (16.4)	0.061ᶜ
Bilateral echogenicity	34 (65.4)	4 (44.4)	152 (59.4)	0.434ᶜ
Respiratory distress syndrome	32 (61.5)	6 (66.7)	127 (49.6)	0.218ᶜ
Sepsis	28 (53.8)	3 (33.3)	79 (30.9)	0.006ᶜ
Disseminated intravascular coagulation	29 (55.8)	4 (44.4)	66 (25.8)	<0.001ᶜ
Congenital heart disease	21 (40.4)	2 (22.2)	77 (30.1)	0.326ᶜ
Necrotizing enterocolitis	22 (42.3)	2 (22.2)	44 (17.2)	<0.001ᶜ
Multiple organ failure	4 (7.7)	0 (0.0)	1 (0.4)	0.008ᶜ

CP, cerebral palsy. Values are median (IQR) or *n* (%), using outcome-group column denominators. Birth weight *n* = 52, 9, and 255; other displayed variables have complete data. “Survival without recorded CP” does not establish normal neurodevelopment. ᵃKruskal–Wallis; ᵇPearson *χ*^2^; ᶜFisher–Freeman–Halton exact; 𝒏ixed-margin Monte Carlo exact test for the entire 3 × 3 Table (200,000 samples; seed 20,260,927; Monte Carlo SE ≈0.00010). Categorical binary rows test the displayed category versus its complement. These exploratory *p* values are not adjusted for multiple comparisons and were not used for covariate screening.

Birth weight (*p* = 0.025) and comorbidity count (*p* < 0.001) differed across the three recorded outcome groups in Kruskal–Wallis analyses; continuous gestational age did not (*p* = 0.189). Exact categorical comparisons showed differences for sepsis (*p* = 0.006), DIC (*p* < 0.001), NEC (*p* < 0.001), and MOF (*p* = 0.008). Multiple birth (*p* = 0.061), sex (*p* = 0.070), delivery mode (*p* = 0.544), laterality (*p* = 0.434), RDS (*p* = 0.218), and CHD (*p* = 0.326) did not reach the nominal significance threshold. These exploratory comparisons do not establish independent effects.

### Adjusted associations with clinical outcomes

In the complete-case mortality model, each additional comorbidity was associated with greater odds of death (adjusted OR, 2.462; 95% CI, 1.787–3.391; *p* < 0.001). Delivery mode, birth weight, gestational age, sex, multiple birth, and laterality did not reach the nominal significance threshold (Table 3). The smaller model yielded a comorbidity OR of 2.348 (95% CI, 1.725–3.195; *p* < 0.001; Supplementary Table S2). These are within-cohort associations, not evidence of causal effects or a validated prediction rule.

**Table 3:** Exploratory multivariable logistic regression for recorded mortality.

Covariate/contrast	*B*	SE	Adjusted OR (95% CI)	*p*
Vaginal vs. cesarean delivery	0.378	0.353	1.459 (0.730–2.916)	0.285
Birth weight per 100 g	−0.002	0.038	0.998 (0.927–1.075)	0.967
Gestational age per week	0.076	0.076	1.079 (0.930–1.252)	0.314
Female vs. male	−0.580	0.367	0.560 (0.273–1.150)	0.114
Multiple vs. singleton birth	−0.839	0.510	0.432 (0.159–1.175)	0.100
Comorbidity score per point	0.901	0.163	2.462 (1.787–3.391)	<0.001
Bilateral vs. unilateral echogenicity	0.232	0.350	1.261 (0.635–2.506)	0.507

*Outcome*: death (52) versus all survivors (264), including survivors with cerebral palsy; complete-case *n* = 316. One infant with invalid birth weight was excluded. All seven listed covariates were entered together. Left- and right-sided lesions were combined as unilateral (reference); cesarean delivery, male sex, and singleton birth were the other categorical references. Birth weight is per 100 g, gestational age per week, and comorbidity count per additional condition. Hospitalization duration was excluded. *B*, log-odds coefficient; SE, standard error; OR, odds ratio; CI, Wald confidence interval. The model describes associations and has not been externally validated.

The recalculated multinomial model also associated comorbidity count with death relative to survival without recorded cerebral palsy (OR, 2.454; 95% CI, 1.780–3.381; *p* < 0.001; *n* = 316). Combining left and right lesions removed the specific zero-event left-sided category, but did not increase the number of cerebral palsy events. The cerebral palsy coefficients remained imprecise and were not interpreted as evidence for or against independent risk factors (Supplementary Table S1).

### White matter abnormalities and cerebral palsy

White matter abnormalities were recorded in nine infants: eight with cerebral palsy and one without recorded cerebral palsy. One of the nine infants with cerebral palsy had no recorded white matter abnormality. None of the 52 infants who died had a positive record, which does not establish that white matter injury was absent before death. Using the derived gestational age groups, one positive record occurred before 30 weeks and eight at 30–36 weeks (Table 4).

**Table 4:** Recorded white matter abnormality indicator by clinical outcome and gestational age.

Recorded outcome	Total	Negative indicator	Positive indicator
Death	52	52 (100.0)	0 (0.0)
Cerebral palsy	9	1 (11.1)	8 (88.9)
Survival without recorded cerebral palsy	256	255 (99.6)	1 (0.4)

Derived gestational age groups			
Gestational age	Total	Negative indicator	Positive indicator
<30 weeks	58	57 (98.3)	1 (1.7)
30–36 weeks	174	166 (95.4)	8 (4.6)
≥37 weeks	85	85 (100.0)	0 (0.0)

Percentages are within rows. Overall, 9/317 infants had a positive indicator and 308/317 had a negative indicator. The original field was named PVL and is reported here as a broad record of white matter abnormalities; it does not imply that all positive records represented cystic PVL. Subtype-specific imaging criteria and detection times were not consistently available. A negative record in an infant who died does not exclude unobserved injury. Counts are descriptive; no causal or adjusted cerebral palsy inference is made.

## Discussion

In this cohort of neonates with isolated subependymal echogenicity on cranial ultrasound, systemic comorbidity burden was associated with mortality, and white matter abnormalities were recorded in eight of nine children with cerebral palsy. MRI findings were compatible with hemorrhage in approximately two-thirds of the selected infants examined. These observations address different aspects of prognosis: the entry ultrasound appearance defines the study population, systemic illness characterizes mortality risk within that population, and later clinical records identify persistent motor disability. The absence of a comparison cohort without the ultrasound finding prevents attribution of these outcomes to subependymal echogenicity itself.

The distinction between echogenicity and confirmed hemorrhage is central to interpreting the imaging results. Residual germinal matrix tissue and other nonhemorrhagic changes can resemble a small hemorrhagic lesion on ultrasound (Ní Leidhin et al., 2025; Indrakanti et al., 2022). Yazol et al. (2026) examined caudothalamic groove echogenicity using complementary MRI, including susceptibility-sensitive imaging, and provide directly relevant evidence for this diagnostic uncertainty. In the present cohort, MRI was selectively obtained and the ultrasound-to-MRI interval varied, with an IQR of 1–30 days. Interval evolution, initially nonhemorrhagic echogenicity, and differences in acquired sequences can each contribute to MRI nonconfirmation. Consequently, the 61/90 proportion describes MRI findings in the examined subgroup; it cannot establish ultrasound specificity or identify the explanation for each negative MRI.

The association between comorbidity count and mortality persisted after adjustment for the included clinical covariates and in the smaller sensitivity model. This finding is consistent with the clinical importance of concurrent systemic disease in neonates with cerebral vulnerability (McQuillen et al., 2010; Ristovska, 2024; Mullaart et al., 1995; Adams-Chapman, 2018). However, the count gives equal weight to conditions with different severity, duration, and overlap. The timing of the complications relative to deterioration and death was not sufficiently characterized to distinguish baseline risk from complications accompanying terminal illness. The observed association should therefore be interpreted as a relationship with recorded illness burden. It neither validates the count for individual prediction nor establishes that comorbidity is causally more important than gestational age, birth weight, or lesion laterality.

Mortality and cerebral palsy also require different analytical interpretations. Hospitalization duration was excluded from the regression because an early death can shorten the admission, making a protective interpretation of longer hospitalization misleading. Comparing death with all survivors retains children with cerebral palsy in the mortality reference group. By contrast, the cerebral palsy proportion among survivors describes children who lived long enough for a diagnosis to be recorded. Death can prevent subsequent neurological assessment, so the two outcomes cannot be treated as independent manifestations of a common follow-up process. Neither the binary model nor the supplementary multinomial model accounts for event timing or estimates a competing-risk cumulative incidence.

The overlap between white matter abnormalities and cerebral palsy warrants clinical attention, but its interpretation depends on both diagnostic specificity and ascertainment. White matter abnormalities were present in eight of the nine recorded cerebral palsy cases, whereas one child with an abnormality had no recorded cerebral palsy. The imaging category therefore did not map exactly onto the clinical diagnosis. Cystic PVL, punctate lesions, and persistent echogenicity differ in their imaging appearance and clinical implications (Mohammad et al., 2021; Ní Leidhin et al., 2025; Dudink et al., 2020); pooling them limits phenotype-specific conclusions. In addition, selective MRI and variable serial imaging can affect which lesions are recognized. With only nine cerebral palsy cases, the findings remain descriptive and do not establish prediction, mediation, or a causal pathway. The study did not directly measure inflammation, cerebral autoregulation, or other proposed mechanisms of injury.

Clarifying outcome ascertainment also changes the interpretation of the follow-up findings. Cerebral palsy is a clinical motor diagnosis, and its documentation in rehabilitation or neurology records provides a different type of evidence from an early developmental score (Rosenbaum et al., 2007; Richards and Malouin, 2013). Bayley-III evaluates early developmental performance; a low cognitive or motor composite score is not, by itself, a diagnosis of cerebral palsy (Balasundaram and Avulakunta, 2022; Robertson, 2010). In this study, the early MDI/PDI and NBNA entries did not define the outcome groups. Telephone follow-up in January 2026 supplemented existing clinical diagnoses but was not a repeat standardized developmental examination. It can help corroborate an established persistent diagnosis, while remaining insufficient to exclude less evident cognitive, language, learning, behavioral, or motor difficulties. Thus, survival without recorded cerebral palsy should not be interpreted as normal long-term neurodevelopment.

These findings support interpreting neonatal imaging together with the clinical course and subsequent neurological assessment. An initially isolated echogenic finding does not establish that later white matter abnormalities or developmental difficulties will be absent. Repeat ultrasound and complementary MRI may help characterize evolving findings, while developmental testing and specialist examination answer different clinical questions (Mohammad et al., 2021; Ní Leidhin et al., 2025; Dudink et al., 2020; Richards and Malouin, 2013). The present study did not compare surveillance strategies and cannot determine an optimal MRI window, a universal ultrasound interval, or a treatment protocol. Prospective studies with standardized imaging definitions and age-appropriate developmental assessments are needed to determine which aspects of follow-up improve prognostic information.

Several limitations should be considered. This was a single-center retrospective study without a comparison cohort lacking the entry ultrasound finding. Ultrasound misclassification, clinically selected MRI, and incomplete acquisition and susceptibility-sequence documentation limit imaging interpretation. Although Bayley-III assessment and subsequent telephone follow-up were undertaken, the present analysis was based on recorded clinical outcomes rather than a standardized score-defined developmental endpoint. Exact cerebral palsy diagnosis ages and clinician identities could not be retrieved, limiting retrospective verification of diagnostic consistency and timing. Telephone follow-up did not provide comprehensive school-age assessment. The mortality analyses remain exploratory, with 52 events, correlated gestational age and birth weight, possible residual confounding, and no external validation. Only nine cerebral palsy events were available, precluding reliable multivariable inference for that outcome. The pooled white matter category lacks sufficiently detailed subtype and timing information. These limitations restrict causal interpretation and comprehensive long-term developmental prognosis.

## Conclusion

Among neonates with isolated subependymal echogenicity on cranial ultrasound, higher recorded systemic comorbidity burden was associated with mortality. White matter abnormalities were recorded in eight of nine children with cerebral palsy, whose diagnoses were ascertained from clinical records and supplemented by later telephone follow-up. The findings support a distinction between early ultrasound appearances, systemic illness burden, and clinically diagnosed motor disability. They do not establish the independent prognostic effect of the ultrasound finding or normal long-term development in survivors without recorded cerebral palsy.

## Data Availability

The raw data supporting the conclusions of this article will be made available by the authors, without undue reservation.

## References

[ref1] Adams-Chapman I. (2018). Necrotizing enterocolitis and neurodevelopmental outcome. Clin. Perinatol. 45, 453–466. doi: 10.1016/j.clp.2018.05.014, 30144849

[ref2] Ahmann P. A. Lazzara A. Dykes F. D. Brann A. W. Jr. Schwartz J. F. (1980). Intraventricular hemorrhage in the high-risk preterm infant: incidence and outcome. Ann. Neurol. 7, 118–124. doi: 10.1002/ana.410070205, 7369717

[ref3] Atienza-Navarro I. Alves-Martinez P. Lubian-Lopez S. Garcia-Alloza M. (2020). Germinal matrix-intraventricular hemorrhage of the preterm newborn and preclinical models: inflammatory considerations. Int. J. Mol. Sci. 21:8343. doi: 10.3390/ijms21218343, 33172205 PMC7664434

[ref4] Balasundaram P. Avulakunta I. D. Bayley scales of infant and toddler development. In StatPearls. Ed. I. D. Avulakunta. Treasure Island (FL): StatPearls Publishing. (2022) Available online at: https://www.ncbi.nlm.nih.gov/books/NBK567715/, (accessed 27 September 2026). 33620792

[ref5] Brew N. Walker D. Wong F. Y. (2014). Cerebral vascular regulation and brain injury in preterm infants. Am. J. Physiol. Regul. Integr. Comp. Physiol. 306, R773–R786. doi: 10.1152/ajpregu.00487.2013, 24647591

[ref6] Dolfin T. Skidmore M. B. Fong K. W. Hoskins E. M. Milligan J. E. Moore D. C. . (1984). Perinatal factors that influence the incidence of subependymal and intraventricular hemorrhage in low birthweight infants. Am. J. Perinatol. 1, 107–113. doi: 10.1055/s-2007-999985, 6518059

[ref7] Dolfin T. Skidmore M. B. Fong K. W. Hoskins E. M. Shennan A. T. (1983). Incidence, severity, and timing of subependymal and intraventricular hemorrhages in preterm infants born in a perinatal unit as detected by serial real-time ultrasound. Pediatrics 71, 541–546. doi: 10.1542/peds.71.4.541, 6835737

[ref8] Dudink J. Jeanne Steggerda S. Horsch S. (2020). State-of-the-art neonatal cerebral ultrasound: technique and reporting. Pediatr. Res. 87, 3–12. doi: 10.1038/s41390-020-0776-y PMC7098885 32218539

[ref9] Fanconi S. Duc G. (1987). Intratracheal suctioning in sick preterm infants: prevention of intracranial hypertension and cerebral hypoperfusion by muscle paralysis. Pediatrics 79, 538–543. doi: 10.1542/peds.79.4.538, 3547301

[ref10] Fichera G. Stramare R. Bisogno G. Wyttenbach R. Goeggel B. S. Del Grande F. . (2024). Neonatal cerebral ultrasound: anatomical variants and age-related diseases. J. Ultrasound 27, 993–1002. doi: 10.1007/s40477-024-00914-8, 38918318 PMC11496415

[ref11] Indrakanti S. Chavez W. Castro-Aragon I. (2022). Normal variant residual germinal matrix in extremely premature infants: radiographic features and imaging pitfalls. J. Ultrasound 25, 493–505. doi: 10.1007/s40477-021-00612-9, 35092600 PMC9402871

[ref12] Lekic T. Klebe D. Poblete R. Krafft P. R. Rolland W. B. Tang J. . (2015). Neonatal brain hemorrhage (NBH) of prematurity: translational mechanisms of the vascular-neural network. Curr. Med. Chem. 22, 1214–1238. doi: 10.2174/0929867322666150114152421, 25620100 PMC4495901

[ref13] McQuillen P. S. Goff D. A. Licht D. J. (2010). Effects of congenital heart disease on brain development. Prog. Pediatr. Cardiol. 29, 79–85. doi: 10.1016/j.ppedcard.2010.06.011, 20802830 PMC2927012

[ref14] Mohammad K. Scott J. N. Leijser L. M. Zein H. Afifi J. Piedboeuf B. . (2021). Consensus approach for standardizing the screening and classification of preterm brain injury diagnosed with cranial ultrasound: a Canadian perspective. Front. Pediatr. 9:618236. doi: 10.3389/fped.2021.618236, 33763394 PMC7982529

[ref15] Moradi B. Ardestani R. M. Shirazi M. Eslamian L. Kazemi M. A. (2024). Fetal intracranial hemorrhage and infarct: main sonographic and MRI characteristics: a review article. Eur. J. Obstet. Gynecol. Reprod. Biol. X 24:100351. doi: 10.1016/j.eurox.2024.100351, 39610469 PMC11603015

[ref16] Mullaart R. A. Hopman J. C. Rotteveel J. J. Daniëls O. Stoelinga G. B. De Haan A. F. . (1995). Influence of end expiratory pressure on cerebral blood flow in preterm infants. Early Hum. Dev. 40, 157–165. doi: 10.1016/0378-3782(94)01603-m, 7750442

[ref17] Ng P. C. Fok T. F. Lee C. H. Wong W. Cheung K. L. (1998). Massive subdural haematoma: an unusual complication of septicaemia in preterm very low birthweight infants. J. Paediatr. Child Health 34, 296–298. doi: 10.1046/j.1440-1754.1998.00220.x, 9633982

[ref18] Ní Leidhin C. Paddock M. Parizel P. M. Warne R. R. Shipman P. Lakshmanan R. (2025). Paediatric cranial ultrasound: assessment of the preterm brain. Insights Imaging 16:158. doi: 10.1186/s13244-025-02030-5, 40696054 PMC12283540

[ref19] Ostrem B. E. L. Gano D. (2025). Novel approaches to the treatment of preterm white matter injury through targeting remyelination. Clin. Perinatol. 52, 289–306. doi: 10.1016/j.clp.2025.02.005, 40350212

[ref20] Richards C. L. Malouin F. (2013). Cerebral palsy: definition, assessment and rehabilitation. Handb. Clin. Neurol. 111, 183–195. doi: 10.1016/b978-0-444-52891-9.00018-x, 23622163

[ref21] Ristovska S. (2024). Respiratory distress syndrome (RDS) in newborns with hypoxic-ischemic encephalopathy (HIE). Pril. (Makedon. Akad. Nauk. Umet. Odd. Med. Nauki.) 45, 19–30. doi: 10.2478/prilozi-2024-0003, 38575384

[ref22] Robertson G. J. (2010). “Bayley scales of infant and toddler development,” in The Corsini Encyclopedia of Psychology, ed. I. D. Avulakunta. Hoboken, NJ: John Wiley & Sons. 1–2.

[ref23] Rosenbaum P. Paneth N. Leviton A. Goldstein M. Bax M. Damiano D. . (2007). A report: the definition and classification of cerebral palsy April 2006. Dev. Med. Child Neurol. Suppl. 109, 8–14. doi: 10.1111/j.1469-8749.2007.tb12610.x, 17370477

[ref24] Serdar M. Walther K. A. Gallert M. Kempe K. Obst S. Labusek N. . (2025). Prenatal inflammation exacerbates hyperoxia-induced neonatal brain injury. J. Neuroinflammation 22:57. doi: 10.1186/s12974-025-03389-4, 40022130 PMC11871844

[ref25] Song I. G. (2023). Neurodevelopmental outcomes of preterm infants. Clin. Exp. Pediatr. 66, 281–287. doi: 10.3345/cep.2022.00822, 36596743 PMC10331553

[ref26] Tuburan S. (2021). “Chapter 18—imaging of the neonate: preterm infant,” in Problem Solving in Pediatric Imaging, eds. Sarvis Milla S. Lala S. (Philadelphia: Elsevier), 355–367.

[ref27] Vesoulis Z. A. Mathur A. M. (2017). Cerebral autoregulation, brain injury, and the transitioning premature infant. Front. Pediatr. 5:64. doi: 10.3389/fped.2017.00064, 28421173 PMC5377300

[ref28] Yazol M. Akhan B. M. Boyunaga O. L. (2026). Does increased echogenicity in the caudothalamic groove always indicate hemorrhage in early infancy? Ultrasound Q. 42:e00736. doi: 10.1097/RUQ.0000000000000736, 41701613

