# Supplementary material for: Imaging findings and clinical outcomes in neonates with isolated subependymal echogenicity on cranial ultrasound: a retrospective cohort study
Source: Front Hum Neurosci. 2026 Oct 28;20:1945002. doi: 10.3389/fnhum.2026.1945002 (PMC13751464; doi:10.3389/fnhum.2026.1945002)
Supplement: Supplementary file 1 [file Table_1.DOCX]

**Supplementary Table S1. Recomputed multinomial model of recorded clinical outcomes**

## Death versus survival without recorded cerebral palsy

| **Covariate / contrast** | **B** | **SE** | **Adjusted OR (95% CI)** | **P** |
| --- | --- | --- | --- | --- |
| Vaginal vs cesarean delivery | 0.342 | 0.355 | 1.408 (0.703–2.820) | 0.335 |
| Birth weight per 100 g | -0.005 | 0.038 | 0.995 (0.923–1.072) | 0.897 |
| Gestational age per week | 0.078 | 0.076 | 1.081 (0.931–1.255) | 0.308 |
| Female vs male | -0.618 | 0.368 | 0.539 (0.262–1.108) | 0.093 |
| Multiple vs singleton birth | -0.784 | 0.513 | 0.457 (0.167–1.248) | 0.127 |
| Comorbidity score per point | 0.898 | 0.164 | 2.454 (1.780–3.381) | <0.001 |
| Bilateral vs unilateral echogenicity | 0.216 | 0.351 | 1.241 (0.623–2.471) | 0.539 |

## Cerebral palsy versus survival without recorded cerebral palsy

| **Covariate / contrast** | **B** | **SE** | **Adjusted OR (95% CI)** | **P** |
| --- | --- | --- | --- | --- |
| Vaginal vs cesarean delivery | -1.304 | 0.826 | 0.272 (0.054–1.369) | 0.114 |
| Birth weight per 100 g | -0.163 | 0.111 | 0.849 (0.684–1.056) | 0.141 |
| Gestational age per week | 0.063 | 0.170 | 1.065 (0.762–1.487) | 0.712 |
| Female vs male | -1.669 | 1.088 | 0.188 (0.022–1.588) | 0.125 |
| Multiple vs singleton birth | 1.068 | 0.760 | 2.909 (0.656–12.890) | 0.160 |
| Comorbidity score per point | -0.121 | 0.359 | 0.886 (0.438–1.790) | 0.735 |
| Bilateral vs unilateral echogenicity | -0.460 | 0.716 | 0.631 (0.155–2.570) | 0.521 |

Complete-case n = 316: 52 deaths, 9 cerebral palsy cases, and 255 survivors without recorded cerebral palsy. All seven covariates were included simultaneously; reference coding and units match Table 3. Hospitalization was excluded. The −2 log likelihood was 303.996; likelihood-ratio χ² = 57.110 (14 df), P < 0.001. This model is retained for comparison with the supplied updated analysis. Only nine cerebral palsy events support that contrast; convergence and merging laterality categories do not make its adjusted estimates reliable. No confirmatory cerebral palsy conclusions are drawn.
