# Supplementary material for: Imaging findings and clinical outcomes in neonates with isolated subependymal echogenicity on cranial ultrasound: a retrospective cohort study
Source: Front Hum Neurosci. 2026 Oct 28;20:1945002. doi: 10.3389/fnhum.2026.1945002 (PMC13751464; doi:10.3389/fnhum.2026.1945002)
Supplement: Supplementary file 2 [file Table_2.DOCX]

**Supplementary Table S2. Smaller mortality sensitivity model**

| **Covariate / contrast** | **Adjusted OR (95% CI)** | **P** |
| --- | --- | --- |
| Gestational age per week | 1.082 (0.989–1.183) | 0.085 |
| Comorbidity score per point | 2.348 (1.725–3.195) | <0.001 |
| Bilateral vs unilateral echogenicity | 1.321 (0.679–2.569) | 0.412 |

n = 317 (52 deaths and 265 survivors). All three covariates were entered together. This model reduces parameter burden and avoids concurrent gestational age and birth weight adjustment. It remains exploratory; it is not a validated prediction model.
